# Supplementary material for: Disulfidptosis: A Novel Prognostic Criterion and Potential Treatment Strategy for Diffuse Large B-Cell Lymphoma (DLBCL)
Source: Int J Mol Sci. 2024 Jun 28;25(13):7156. doi: 10.3390/ijms25137156 (PMC11241771; doi:10.3390/ijms25137156)
Supplement: Supplementary file 1 [file ijms-25-07156-s001.zip › ijms-3026433 Supplementary figures.pdf]

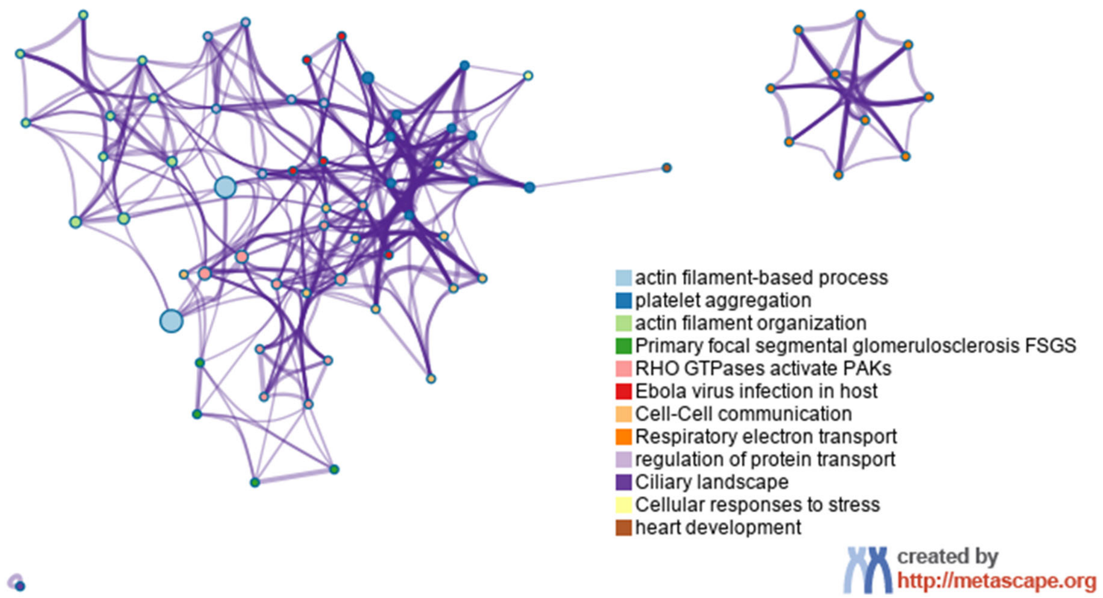

Figure S1. pathway-pathway network of DRGs

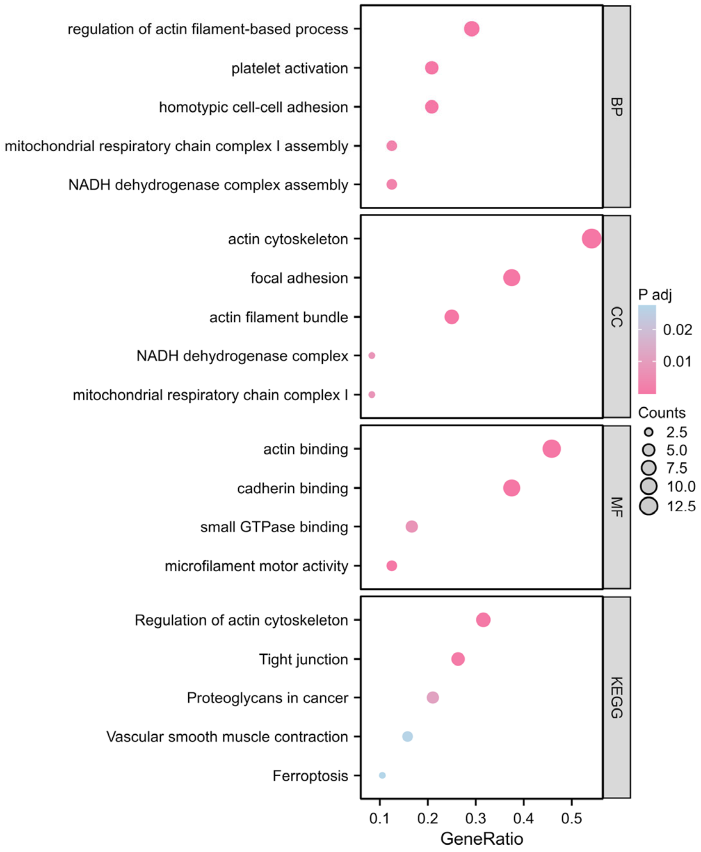

Figure S2. GeneRatio of top pathway of DRGs in DLBCL

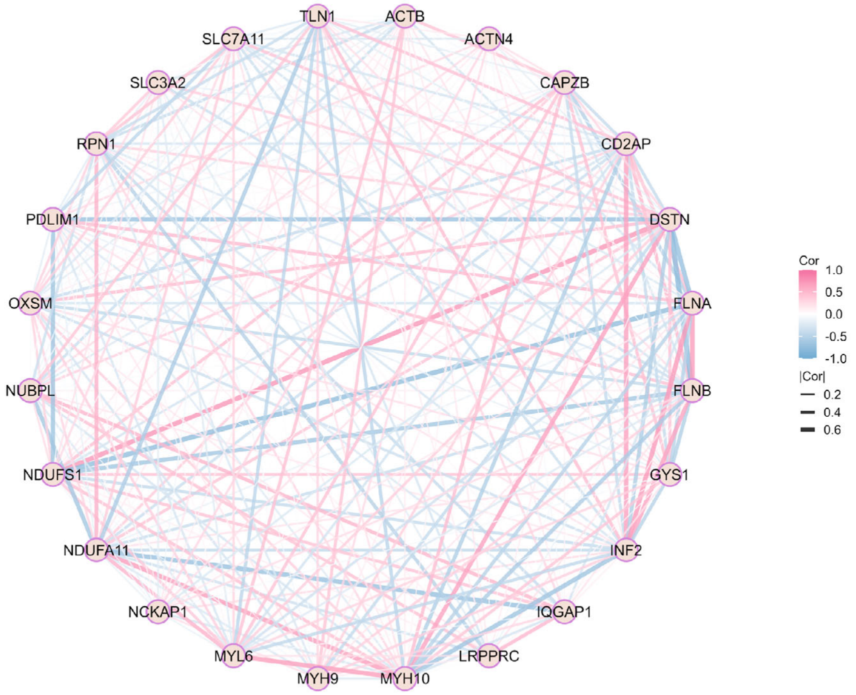

Figure S3. Pearson correlation of the transcriptome relationships in DLBCL

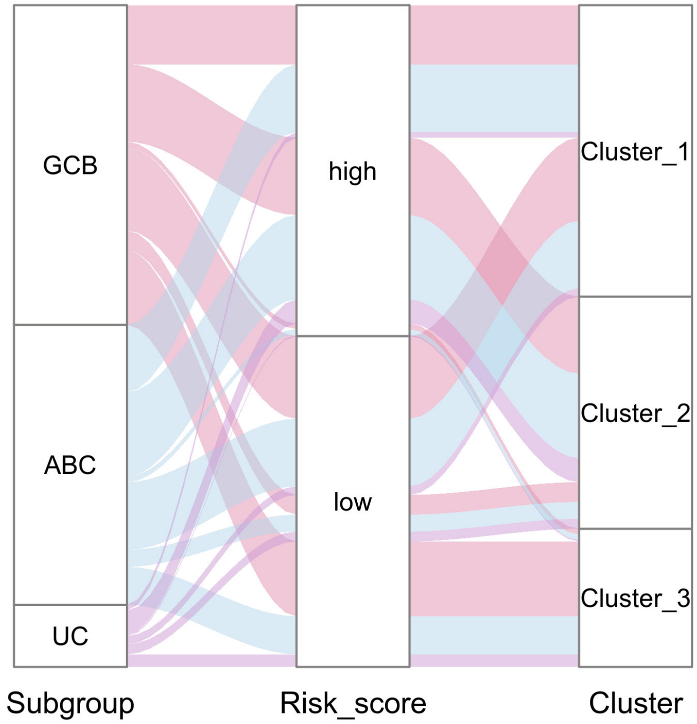

Figure S4. DLBCL subtypes composition of each cluster and the level of risk score demonstrated by the Sankey diagram

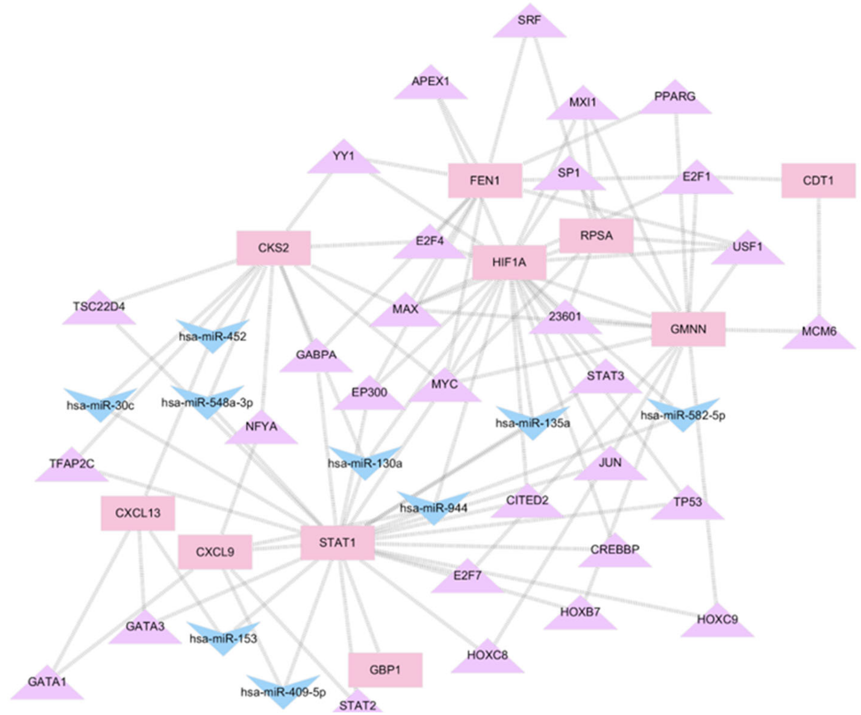

Figure S5. The hub genes-TF-ncRNA interaction network in DLBCL, the pink frame present hub genes, purple triangles present TF, light blue present non-coding RNA

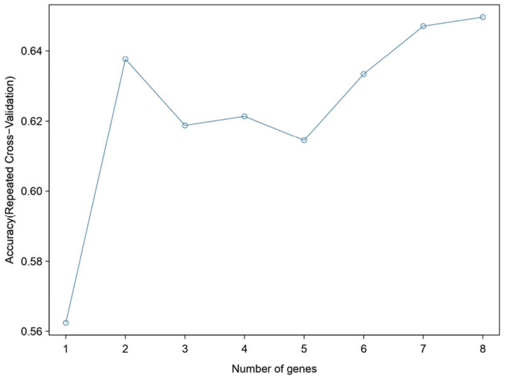

(**A**)

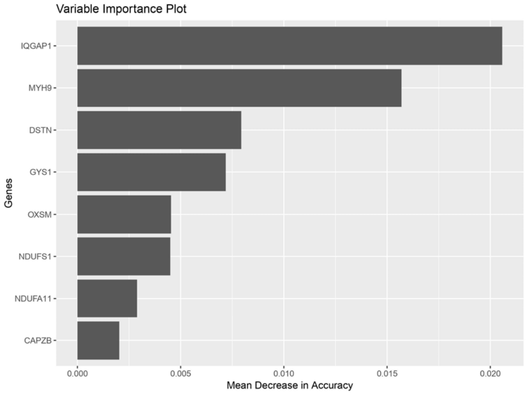

(**B**)

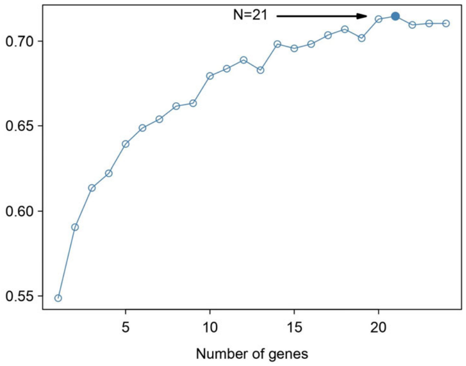

(**C**)

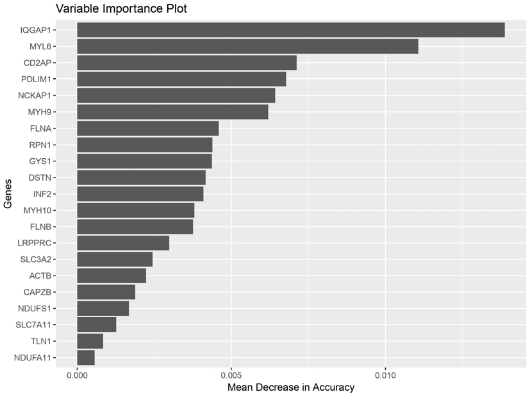

(**D**)

Figure S6. The random forest algorithm, (**A**) and (**C**) show the relationship between predicted accuracy and the number of selected genes(A, 8 DRGs; C, 24-DRGs). (**B**) and (**D**) present the ranking by mean decreasing accuracy (MDA) values in random forest analysis.
